# Microbe Profile: *Ruminococcus gnavus*: the yin and yang of human gut symbionts

**DOI:** 10.1099/mic.0.001383

**Published:** 2023-08-25

**Authors:** Nathalie Juge

**Affiliations:** ^1^​ Quadram Institute Bioscience, Food, Microbiome and Health Institute Strategic Programme, Norwich NR4 7UQ, UK

**Keywords:** gut adaptation, gut microbiota, host–microbe interaction, intestinal and extra-intestinal diseases, intestinal mucus, metabolites, *Ruminococcus gnavus*

## Abstract

*
Ruminococcus gnavus* is a human gut symbiont, part of the infant and adult gut microbiota and associated with intestinal and extra-intestinal disorders. *R. gnavus* mechanisms of adaptation to the gut are strain-specific and underpinned by the capacity of *R. gnavus* strains to utilize mucin and dietary glycans and produce bacteriocins and adhesins. Several potential mediators underpinning the association between *R. gnavus* strains and diseases have been identified, including the capacity to elicit a pro- or anti-inflammatory host response and modulate host metabolism, secondary bile acids and tryptophan metabolic pathways. Based on increasing evidence from metagenomics studies in humans and functional investigations *in vitro* and in mouse models, *R. gnavus* is emerging as a main player in influencing health and disease outcomes from infants to the elderly.

## Graphical abstract

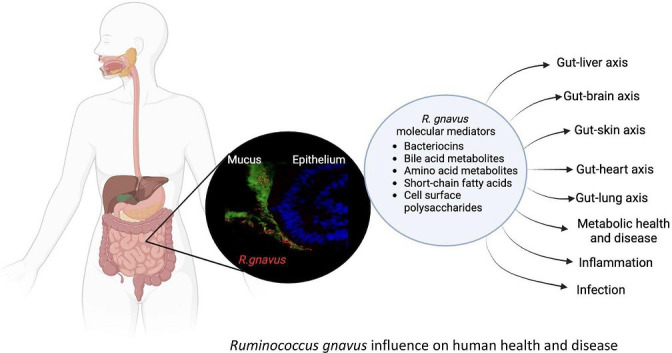

## Taxonomy and properties

*Ruminococcus gnavus* is a Gram-positive anaerobic bacterium belonging to the phylum Firmicutes, the class Clostridia and the cluster XIVa, and the family *Lachnospiraceae *. While retaining its full name from its original classification in the genus *Ruminoccocus* [1], *R. gnavus* has been temporarily classified into the genus *Blautia* and more recently into the genus *Mediterraneibacter* [2]. The representative strain ATCC 29149 (=VPI C7-9) was isolated from human faeces. *R. gnavus* is generally regarded as a non-spore-former and strict anaerobe. *R. gnavus* end-fermentation products of carbohydrates include acetate, formate and ethanol, as well as propanol and propionate but not butyrate [3].

## Occurrence in the human gut microbiota and association with health and disease

*R. gnavus* is a prevalent human gut symbiont and an early colonizer of the infant gut that persists throughout adulthood as 1 of 57 bacterial species present in more than 90 % of individuals with a median abundance of ~0.1 % across populations [4]. *R. gnavus* belongs to the 24 ‘age-discriminatory’ taxa whose changes in relative abundance over time are associated with normal ‘maturation’ of the microbiota and weight gain and metabolism in infants [5].

In addition of being a member of the ‘normal’ microbiota with beneficial properties, an increasing number of intra- and extra-intestinal diseases have been associated with *R. gnavus *, as recently compiled [3], illustrating the yin and yang properties of *R. gnavus* strains. For example, a positive association has been reported with inflammatory bowel disease, including Crohn’s disease, ulcerative colitis, irritable bowel syndrome or colon cancer, and with several features of metabolic syndromes in humans, with a consistently increased proportion of obesity, type 2 diabetes, liver and cardiovascular diseases.

## Key features and properties

The adaptation of *R. gnavus* to the gut is strain-dependent and associated with its capacity to utilize some human milk oligosaccharides and forage on mucin glycan epitopes, such as sialic acid (Neu5Ac), fucose, or blood group antigens in the mucus niche [3]. Notably, *R. gnavus* ATCC 29149 has a unique sialic acid metabolic pathway dedicated to 2,7-anhydro-Neu5Ac [6].

There is a large degree of strain variation in *R. gnavus* functional properties. *R. gnavus* E1 produces several bacteriocins called ruminococcins that are active against pathogenic clostridia and multidrug-resistant strains [7]. Several pro-inflammatory and anti-inflammatory factors have been identified in *R. gnavus* strains such as glucorhamnan and large capsular polysaccharides [8]. This strain variation in the capacity of *R. gnavus* to elicit anti- or pro-inflammatory responses is reflected in a mouse model of diseases showing a beneficial or detrimental outcome of *R. gnavus* supplementation (as reviewed in [3]). At the molecular level, some *R. gnavus* strains display β-d-glucuronidase activity resulting in the removal of glucuronic acid from liver-produced β-d-glucuronides, while others produce an enzyme decarboxylating tryptophan into tryptamine [9] or 7β-hydroxysteroid dehydrogenases generating ursodeoxycholic acid from primary bile [10]. *R. gnavus* tryptophan and bile acid metabolites have been implicated in the role of *R. gnavus* in inflammation and metabolic disorders, but also the development of neuro/psychological disorders [11], although causality remains to be demonstrated.

## Open questions

Does the capacity of *R. gnavus* strains to utilize different carbohydrates *in vitro* reflect ecological niches in the gut?

What is the spatial distribution of *R. gnavus* strains in the infant and adult gastrointestinal tract?

Which *R. gnavus* strains/niches are associated with specific diseases?

Can we rationally design nutritional interventions that target particular *R. gnavus* niche-associated strains?

Which *R. gnavus* functional properties play a causal role in health or diseases?

Can microbial-based approaches such as phage therapy can be used to reduce overgrowth of *R. gnavus* strains associated with diseases while preserving *R. gnavus* associated with host health benefits?

## References

[1] Moore WEC, Johnson JL, Holdeman LV (1976). Emendation of *Bacteroidaceae* and *Butyrivibrio* and descriptions of *Desulfomonas* gen. nov. and ten new species in the genera *Desulfomonas*, *Butyrivibrio*, *Eubacterium*, *Clostridium*, and *Ruminococcus*. Int J Syst Bacteriol.

[2] Togo AH, Diop A, Bittar F, Maraninchi M, Valero R (2018). Description of *Mediterraneibacter massiliensis*, gen. nov., sp. nov., a new genus isolated from the gut microbiota of an obese patient and reclassification of *Ruminococcus faecis*, *Ruminococcus lactaris*, *Ruminococcus torques*, *Ruminococcus gnavus* and *Clostridium glycyrrhizinilyticum* as *Mediterraneibacter faecis* comb. nov., *Mediterraneibacter lactaris* comb. nov., *Mediterraneibacter torques* comb. nov., *Mediterraneibacter gnavus* comb. nov. and *Mediterraneibacter glycyrrhizinilyticus* comb. nov. Antonie van Leeuwenhoek.

[3] Crost EH, Coletto E, Bell A, Juge N (2023). *Ruminococcus gnavus*: friend or foe for human health. FEMS Microbiol Rev.

[4] Qin J, Li R, Raes J, Arumugam M, Burgdorf KS (2010). A human gut microbial gene catalogue established by metagenomic sequencing. Nature.

[5] Blanton LV, Charbonneau MR, Salih T, Barratt MJ, Venkatesh S (2016). Gut bacteria that prevent growth impairments transmitted by microbiota from malnourished children. Science.

[6] Bell A, Brunt J, Crost E, Vaux L, Nepravishta R (2019). Elucidation of a sialic acid metabolism pathway in mucus-foraging *Ruminococcus gnavus* unravels mechanisms of bacterial adaptation to the gut. Nat Microbiol.

[7] Roblin C, Chiumento S, Bornet O, Nouailler M, Müller CS (2020). The unusual structure of Ruminococcin C1 antimicrobial peptide confers clinical properties. Proc Natl Acad Sci.

[8] Henke MT, Brown EM, Cassilly CD, Vlamakis H, Xavier RJ (2021). Capsular polysaccharide correlates with immune response to the human gut microbe *Ruminococcus gnavus*. Proc Natl Acad Sci U S A.

[9] Williams BB, Van Benschoten AH, Cimermancic P, Donia MS, Zimmermann M (2014). Discovery and characterization of gut microbiota decarboxylases that can produce the neurotransmitter tryptamine. Cell Host Microbe.

[10] Lee J-Y, Arai H, Nakamura Y, Fukiya S, Wada M (2013). Contribution of the 7β-hydroxysteroid dehydrogenase from *Ruminococcus gnavus* N53 to ursodeoxycholic acid formation in the human colon. J Lipid Res.

[11] Coletto E, Latousakis D, Pontifex MG, Crost EH, Vaux L (2022). The role of the mucin-glycan foraging *Ruminococcus gnavus* in the communication between the gut and the brain. Gut Microbes.

